# Spatial distribution characteristics of plumes induced by femtosecond laser ablation of silicon in vacuum

**DOI:** 10.1038/s41598-023-33933-2

**Published:** 2023-04-24

**Authors:** Zhandong Chen, Hua Ning, Xiulan Zhang

**Affiliations:** School of Mathematics and Physics, Guangxi Minzu University, Nanning, 530006 China

**Keywords:** Laser material processing, Optical spectroscopy

## Abstract

The spatial distribution characteristics of plumes induced by femtosecond laser ablation of silicon in vacuum are studied by using spectroscopy. The plume spatial distribution clearly shows two zones with different characteristics. The center of the first zone is at a distance of approximately 0.5 mm from the target. Silicon ionic radiation, recombination radiation, and bremsstrahlung mainly occur in this zone, causing an exponential decay with a decay constant of approximately 0.151–0.163 mm. The second zone with a greater area, whose center is at a distance of approximately 1.5 mm from the target, follows the first zone. In this zone, the radiation from silicon atoms and electron-atom collisions dominates, leading to an allometric decay with an allometric exponent of approximately − 1.475 to − 1.376. In the second zone, the electron density spatial distribution is approximately arrowhead-shaped, which is potentially induced by collisions between ambient molecules and the particles in front of the plume. These results indicate that both the recombination effect and expansion effect play important roles and compete with each other in plumes. The recombination effect is dominant near the silicon surface, causing exponential decay. As the distance increases, the electron density decreases exponentially by recombination, causing a more intense expansion effect.

## Introduction

In past decades, great improvement has been achieved in the field of femtosecond laser processing of solids, which has been an effective method to prepare new kinds of materials. Femtosecond laser can be used to process various kinds of materials, including metals ^1–4^, semiconductors^5–8^, and dielectrics^9–11^. Femtosecond laser processing can achieve micro-structures^12–14^ and doping^15–17^ on solid surfaces. When the fluence of the femtosecond laser pulse is higher than the ablation threshold of the material, a large number of materials are ejected from the sample surface, forming a plume. As a result, microstructures are produced on the sample surface, leading to novel properties. A large area of microstructures with supersaturated doping has been fabricated on silicon surface^18^, resulting in high light absorption^19^. Microstructured silicon is a promising material for the field of solar cells and photoelectric detection^20^. During femtosecond laser irradiation, the energy of the laser pulse is deposited onto the sample surface on a femtosecond time scale, causing an extremely high energy density. The relaxation of the energy dominates the morphology of the surface microstructure and the formation of the plume. A better understanding of the mechanism of energy relaxation and material removal will be helpful to improve femtosecond laser processing technology. Much research has been devoted to studies on the dynamics of femtosecond laser-induced plumes by means of various methods, such as theoretical simulation^21,22^, time-resolved fast imaging^23,24^, ultrafast time-resolved microscopy^25^, optical emission spectroscopy^26–29^, and time-of-flight (TOF) mass spectroscopy^30^. However, the formation and evolution of plumes near the sample surface are very complex, causing difficulties in obtaining a deeper understanding of the material removal mechanism during femtosecond laser ablation.

In this study, the spatial distribution characteristics of the plume induced by femtosecond laser ablation of silicon in vacuum are studied by using spectroscopy. The species in the plume are determined by the spectral lines emitted from the silicon atoms and monovalent ions. The spatial intensity distributions of different spectral components are also discussed. Two evolution mechanisms are confirmed by introducing a new function to fit the experimental data. The Voigt function is used to fit all spectral lines and obtain their full width at half maximum (FWHM). Based on the FWHM of the lines, the electron density of the plume is derived by using the Stark-broadening method. The two-peak structure of the electron density distribution is observed and studied in detail. Based on the experimental results, the effects of recombination and plume expansion are fully discussed to provide deeper insight into the spatial distribution characteristics of femtosecond laser induced plumes.

## Experiments

In this study, a Ti:sapphire regenerative amplifier, which delivers 120-fs laser pulses with a repetition rate of 1 kHz at a central wavelength of 800 nm, is used. The laser pulses are focused by a lens with a focal length of 0.5 m, pass through a quartz window and then propagate into a vacuum chamber. The laser pulses are directed perpendicularly onto an n-type silicon (100) wafer with a resistivity of more than 3000 Ωcm. The diameter of the light spot on the wafer’s surface is 63 μm, causing a fluence of 4 J/cm^2^. The silicon wafer is immersed in hydrofluoric acid for 15 min to remove the native oxide and then rinsed with distilled water; it is then mounted on a three-axis translation stage in the vacuum chamber with a base pressure less than 1 Pa. The stage is set to move in the x–y plane with a speed of 0.5 mm/s.

**Figure 1:**
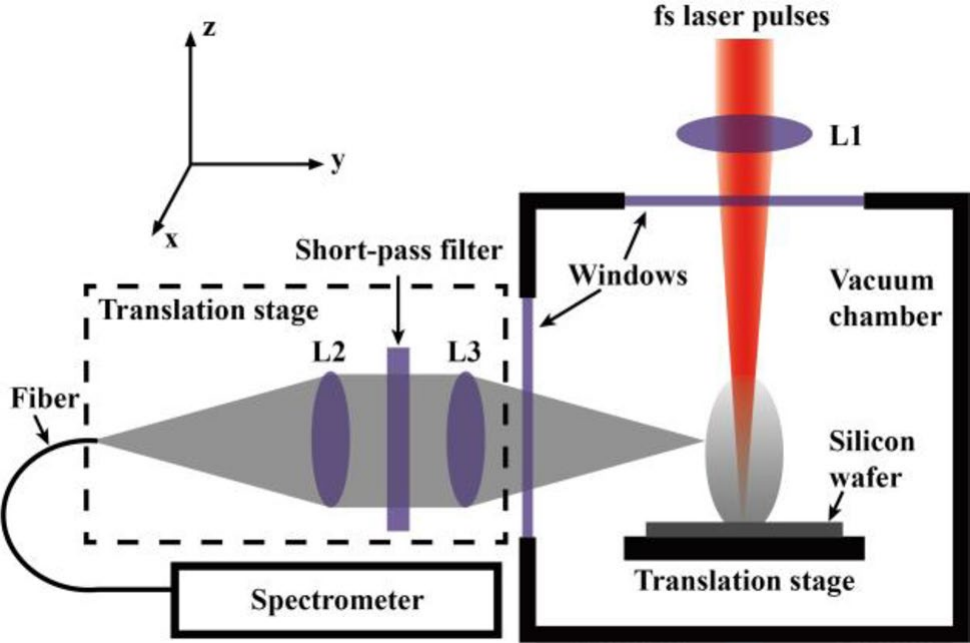
Schematic of the experimental setup.

A light collection system, which consists of two quartz lenses and a short-pass filter, is mounted on a three-axis translation stage in the lateral direction and used to collect the emission light of the plume. The collected light is focused on a quartz fiber that is connected to a spectrometer (HR4000CG-UV-NIR, Ocean Optics). The light collection system with a spatial resolution of 200 μm is moved in the x–z plane by a step of 200 μm such that the emission spectra of the whole plume are detected point by point. The experimental setup is shown in Fig. 1. In this case, the time-averaged emission spectra of the plasma plume are recorded and the obtained plasma spatial distributions are also time-averaged. Moreover, the results indicate some interesting properties of femtosecond laser-induced plasma plumes.

## Results and discussion

**Figure 2:**
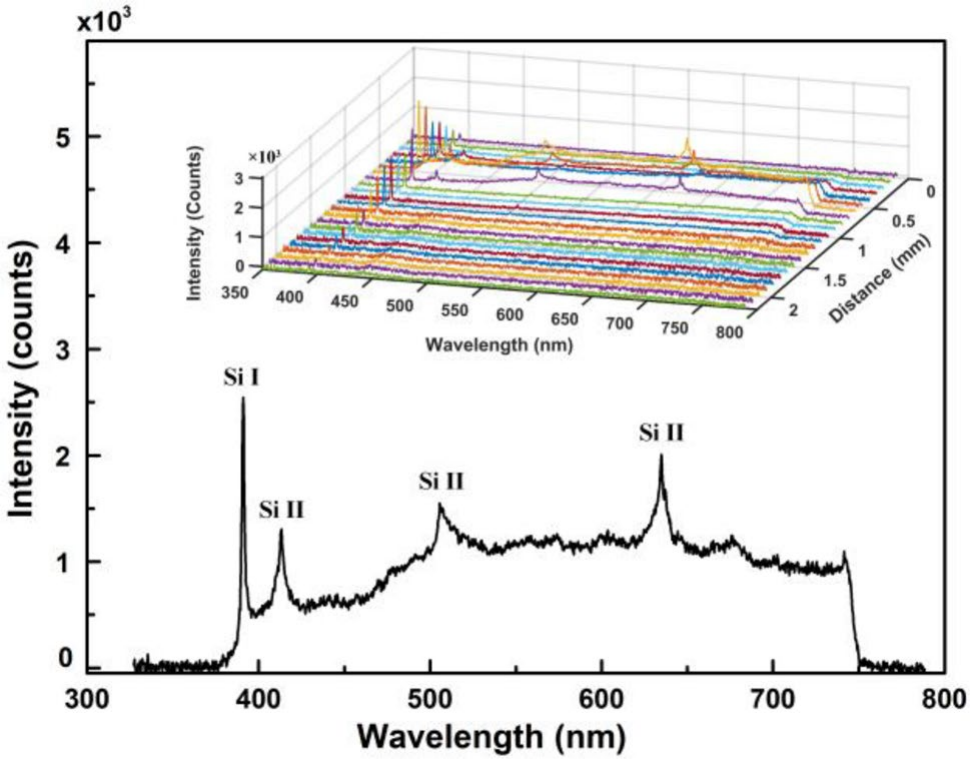
The spectrum of the plume at x = 0 and z = 0.6 mm. Inset: spectra at different distances along the z-axis.

**Figure 3:**
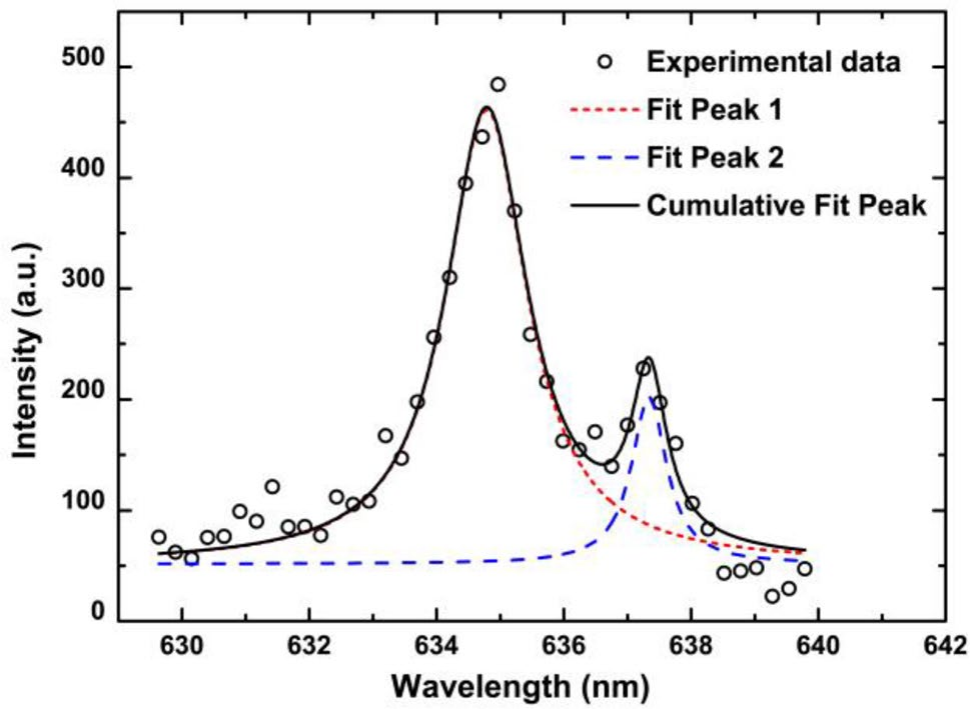
Fittings of two peaks for the Si II lines at approximately 635 nm with the Voigt function.

The emission spectra of the fs-laser-induced plume on the silicon surface are detected in the x–z plane. All spectral data are corrected for the response of the detecting system. Figure 2 shows the corrected spectra of the plume at x = 0 and z = 0.6 mm. Beneath several spectral lines radiated by Si I and Si II, there is a continuous spectrum (CS), which is likely caused by bremsstrahlung, recombination, and electron-atom collisions. The Si II line at approximately 635 nm can be separated into two peaks, as shown in Fig. 3. The spectral lines are confirmed according to the NIST Atomic Spectra Database^31^, as shown in Table 1. The silicon atoms and silicon monovalent ions are two of the main components in the fs-laser-induced plume. The inset in Fig. 2 shows the spatial evolution of the spectra along the z-axis, which indicates that the spatial evolution characteristics are different for the silicon atom, the silicon ion, and continuous radiation.

**Table 1:** Information on the spectral lines.

Experimental Wavelength (nm)	Theoretical Wavelength (nm)	Ion	A_ki_ (/s)	E_k_ (/cm)	g_k_
391.03	390.55	Si I	1.33e7	40 991.88	3
413.32	413.09	Si II	1.74e8	103,556.03	8
505.66	505.60	Si II	1.45e8	101,024.35	6
634.79	634.71	Si II	5.84e7	81,251.32	4
637.34	637.14	Si II	6.80e7	81,191.34	2

**Figure 4:**
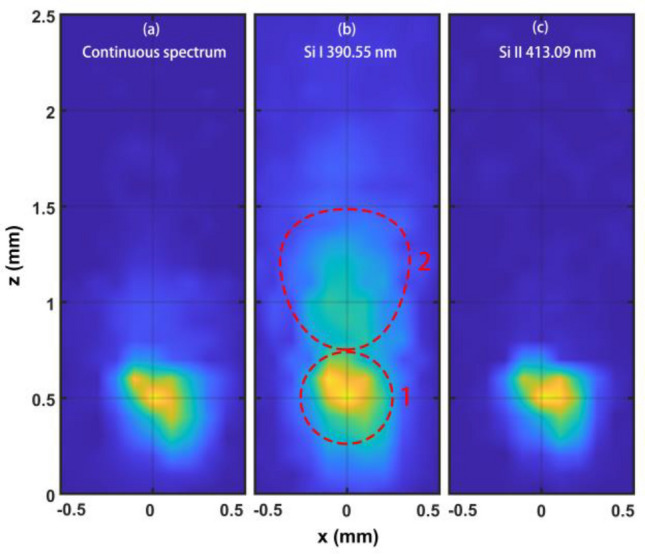
The spatial distribution of the spectral intensity in the plume. (**a**) Continuous spectrum, (**b**) Si I line at 390.55 nm, (**c**) Si II line at 413.09 nm. Each image is normalized to its own maximum intensity.

The spectral lines are obtained by subtracting the continuous spectrum as a baseline. Figure 4 shows the spatial distributions of the spectral intensities of CS, the Si I line at 390.55 nm, and the Si II line at 413.09 nm. The distribution property is similar for other Si II lines. The results indicate that there are two zones with different radiation characteristics. The center of the first zone with a radius of approximately 0.25 mm is approximately at x = 0 and z = 0.5 mm. The continuous radiation, silicon atomic radiation, and silicon ionic radiation are all intense in this zone. The area of silicon atomic radiation is slightly larger than that of continuous radiation and ionic radiation. The radiation from silicon ions is almost absent out of this zone, which means that the silicon ions are mainly produced in the first zone. As a result, the plasma density is very high in this zone, causing very intense continuous radiation, as shown in Fig. 4a. The second zone, which is approximately fan-shaped and behind the first zone, is larger. In the second zone, the radiation from the silicon atoms is considerable and decreases with the distance from the silicon surface. The intensity of the Si I line is still observable at z = 2.5 mm. Unlike atomic radiation, the continuous radiation, which is very weak in the second zone, decreases with increasing distance and becomes unmeasurable at approximately z = 1.4 mm. The nature of the two radiation zones in the fs-laser-induced plume clearly demonstrates that the different physical mechanisms dominate in the two zones. After the fs-laser pulses are focused on the surface, a plasma plume caused by the Coulomb explosion^32^, mainly consisting of atoms, ions, and electrons, is ejected from the sample surface at approximately tens to hundreds of nanoseconds after the laser pulse^20^. Due to the high electron density of the plasma plume, bremsstrahlung radiation and recombination radiation are very intense, leading to strong continuous light emission in the first zone. Moreover, the density of the excited silicon atoms and ions is very high in this zone, resulting in intense light emission from these particles. The electron density decreases quickly with increasing distance through recombination and expansion, causing an absence or a decrease in the ionic radiation, bremsstrahlung radiation, and recombination radiation out of the first zone. Due to recombination, there are very few silicon ions in the second zone. Moreover, the silicon atom density and plume temperature decrease with increasing distance due to plume expansion and light radiation, causing a decrease in silicon atomic radiation in the second zone. In addition, the weak continuous light radiates from the plume by electron-atom collisions in the second zone, as shown in Fig. 4a.

**Figure 5:**
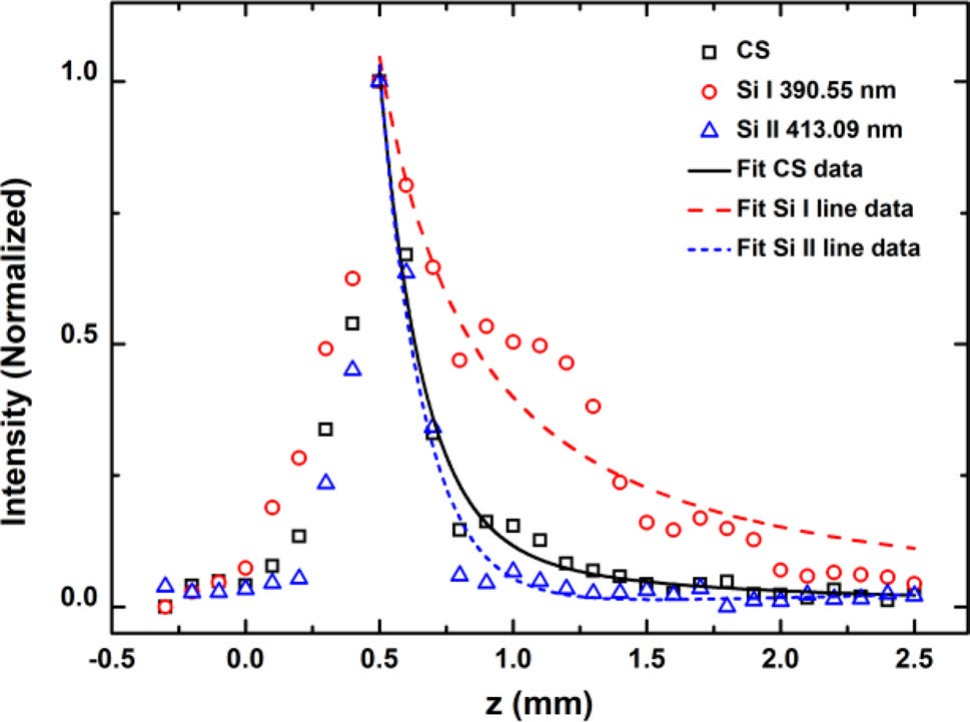
The spatial intensity distribution of CS, Si I line at 390.55 nm, and Si II line at 413.09 nm. The scatter dots are the experimental data, and the lines represent the EA fitting.

To gain deeper insight into the spatial evolution of the radiation intensity in the laser-induced plume, the intensities of the CS, Si I line at 390.55 nm, and Si II line at 413.09 nm are plotted as a function of distance (z) in Fig. 5. The intensity of the spectrum reaches a maximum value at z = 0.5 mm and then decays with increasing distance. Clearly, the decay properties of different spectral components vary. The intensity of the Si II line decays very fast with increasing distance. It can be effectively fitted by an exponential decay function. The decay is slightly slower for CS and much slower for the Si I line, leading to a deviation from the exponential decay profile. The evolution of the plume also depends on the plume expansion effect, which can be approximately described by a simple power law. Therefore, a function that combines the exponential decay function and the allometric function is introduced to fit this type of decay profile. In our study, it is tentatively called the “Exponent-Allometric function” (abbreviation: EA function), as shown below:

1 $$I = A \cdot \exp \left( { - {\raise0.7ex\hbox{$z$} \!\mathord{\left/ {\vphantom {z t}}\right.\kern-0pt} \!\lower0.7ex\hbox{$t$}}} \right) + b \cdot z^{c}$$

where $A$ and $b$ are the factors of the two components that present the contributions of the two decay mechanisms to some extent, and t and c denote the decay speed of the two components. The data from the CS, Si I line, and Si II line are effectively fitted by the EA function, as shown in Fig. 5. The values of the fitting parameters are listed in Table 2. The decay constants $t$ (0.151–0.163 mm) and $c$ (− 1.475 to − 1.376) are nearly the same for the three spectral components. This confirms that there are two evident decay mechanisms. The first one follows an exponential decay law, while the second one follows the allometric decay law. The exponential decay component with a decay constant $t$ of 0.151–0.163 mm leads to a fast decay from z = 0.5 mm to z = 0.9 mm, while the allometric exponent $c$ with a value of − 1.475 to − 1.376 causes a slower decay. The factors $A$ and $b$ for the Si II line are 22.051 and 0.007, respectively, meaning that the exponential decay mechanism is mainly predominant and the other mechanism can be neglected. The intensity of the Si II line radiated from silicon monovalent ions mainly depends on the character of the plasma plume in the first zone. In local thermodynamic equilibrium (LTE), based on the Boltzmann distribution, the intensity of the spectral line is given by the following:

2 $$I = \frac{hc}{{4\pi \lambda }}N\frac{gA}{Z}\exp \left( { - \frac{E}{kT}} \right)$$

where $h$ is the Planck constant, $c$ is the speed of light, $N$ is the upper level population, $Z$ is the partition function,$g$ is the statistical weight, $A$ is the transition probability, $k$ is the Boltzmann constant, $T$ is the absolute temperature, and $E$ is the excitation energy. The intensity of the spectral line depends on both the particle density $N$ and plasma temperature $T$. Due to recombination, the silicon ion density *N* decreases very quickly with increasing distance. Based on the fact that the spatial decay profile can be approximately described by an exponential decay function for the Si II line, the relationship between ion density *N* and distance z should be written as follows:

3 $$N \propto \exp \left( { - \frac{z}{{t_{1} }}} \right)$$

**Table 2:** EA fitting results of different spectral components.

	$A$	$t$ (mm)	$b$	$c$
CS	19.648	0.155	0.085	− 1.475
Si I 390.55 nm	1.477E−15	0.151	0.399	− 1.392
Si II 413.09 nm	22.051	0.163	0.007	− 1.376

The plasma temperature $T$ decreases with increasing distance through plasma expansion and light radiation. The relationship between plasma temperature $T$ and distance z can be approximately written as follows:

4 $$T \propto \frac{1}{z}$$

As a result, the intensity of the Si II line can be written as follows:

5 $$I \propto \exp \left( { - \frac{z}{{t_{1} }}} \right)\exp \left( { - \frac{E \cdot z}{{a \cdot k}}} \right) = \exp \left( { - \frac{z}{t}} \right)$$

where $a$ is the scale factor and $t = \left( {\frac{1}{{t_{1} }} + \frac{E}{a \cdot k}} \right)^{ - 1}$.

Unlike the Si II line, the factors $A$ and $b$ for the Si I line are 1.477 × 10^–15^ and 0.399, respectively, indicating that the allometric decay component is more dominant. This means that the expansion effect determines the evolution of the silicon atom in the plume. In addition, the recombination of the silicon ions causes an increase in silicon atoms, resulting in another intensity peak of the Si I line at z = 0.9 mm, as shown in Fig. 5. The superposition of the expansion effect and recombination effect causes an extremely complex spatial evolution of the silicon atoms. However, the decay profile of the Si I line intensity is generally closer to an allometric decay function.

The factors $A$ and $b$ for CS are 19.648 and 0.085, respectively, indicating that the two decay components are both important for the CS decay characteristics. The exponential decay component with a decay constant of $t$ = 0.155 mm represents the fast decay process of the plasma plume, radiating continuous spectra by recombination and bremsstrahlung in the first zone. The allometric decay component with an allometric exponent of $c$ = − 1.475 denotes the decay process of plume expansion, leading to a slow decay process of continuous spectra caused by electron-atom collisions in the second zone.

In brief, there are two evolution mechanisms in laser-induced plumes. One is the exponential decay component, which represents the evolution characteristic of the plasma plume (in the 1st zone) and is governed by recombination, expansion, and light radiation. The other component is the allometric decay component, which represents the evolution characteristic of the nearly neutral plume (in the 2nd zone) and is mainly governed by the expansion effect.

Due to the high density and high temperature of the laser-induced plasma plume, the most important line-broadening mechanism for the spectral lines is Stark broadening, causing a Lorentzian line profile. The Doppler broadening, which causes a Gaussian line profile, is very small and negligible in our study. In addition, the instrument response of the spectrometer usually causes a Gaussian line profile, which suffices together with the Stark broadening to make the Voigt function, as shown below^33^:

6 $$f\left( \lambda \right) = A\frac{2\ln 2}{{\pi^{{{\raise0.7ex\hbox{$3$} \!\mathord{\left/ {\vphantom {3 2}}\right.\kern-0pt} \!\lower0.7ex\hbox{$2$}}}} }}\frac{{w_{l} }}{{w_{g}^{2} }} \cdot \int_{ - \infty }^{\infty } {\frac{{e^{{ - t^{2} }} }}{{\left( {\sqrt {\ln 2} \frac{{w_{l} }}{{w_{g} }}} \right)^{2} + \left( {\sqrt {4\ln 2} \frac{{\lambda - \lambda_{0} }}{{w_{g} }} - t} \right)^{2} }}} dt$$

where $w_{l}$ and $w_{g}$ are the FWHM of the Lorentzian profile and the Gaussian profile, respectively, and $A$ is the intensity of the line.

**Figure 6:**
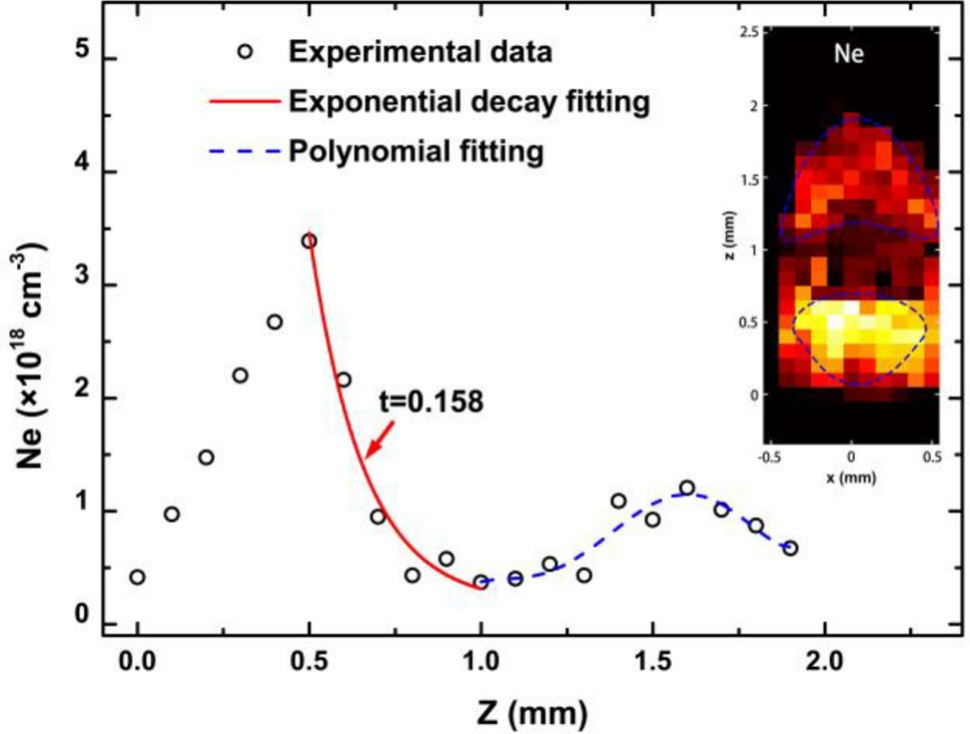
The electron density as a function of distance z; the inset shows the spatial distribution of the electron density in the plume.

The Gaussian profile is mainly caused by the the instrument response of the spectrometer that is almost steady. In this study, the FWHM of the the instrument response function is 0.75 nm. Therefore, the $w_{g}$ in the Voigt function is fixed at 0.75 nm during fitting process. And then, all spectral lines are fitted by the Voigt function to obtain the FWHM $w_{l}$ of the Lorentzian profile. The FWHM $w_{l}$ of a Stark-broadened line is given by the relationship, as shown below^34^:

7 $$w_{l} = 2w_{0} \left( {\frac{{N_{e} }}{{10^{16} }}} \right)$$

where $w_{0}$ is the electron-impact half width with a value of $1.46 \times 10^{ - 13} \;\;{\text{nm}}$ for the Si I line at 390.55 nm^34^. The FWHM $w_{l}$ of the spectral line (Si I line at 390.55 nm) is used to calculate the electron density. The spectral line intensity is very low far from the silicon’s surface, leading to a large uncertainty. Therefore, data with a sufficiently high signal-to-noise ratio (SNR) are selected to plot the electron density spatial distribution, as shown in the inset of Fig. 6. Notably, there are two evident separate zones. The first one, whose center is at x = 0 and z = 0.5 mm, is approximately fan-shaped. The second one at a distance of approximately 1.5 mm is arrowhead-shaped. Figure 6 shows the evolution of the electron density along the z-axis. The value of the first peak is approximately 3.7 × 10^18^/cm^3^, and it is approximately 1.1 × 10^18^/cm^3^ for the second one. The decay curve can be separated into two sections. The first section from z = 0.5 mm to z = 1 mm is effectively fitted via an exponential decay function. The decay constant t is approximately 0.158, which is almost the same as the exponential decay component of the spectral intensity, as shown in Table 2. This indicates that the evolution of the plasma plume in the first zone is governed by an exponential decay mechanism, leading to exponential decay characteristics of the electron density, ion radiation, recombination radiation, and bremsstrahlung radiation. The second section in the decay profile is interesting and simply fitted via a polynomial function. Based on the arrowhead shape of the second zone shown in the inset of Fig. 6, it is reasonable that the increase in the electron density in this zone is probably attributed to the collision between the ambient molecules and the particles in front of the plume. The collision ionization in this zone, which increases the electron density, reaches a peak at a distance of approximately 1.5 mm due to plume expansion that compresses the ambient gas (approximately 1 Pa).

## Conclusions

The spatial distribution characteristic of the plume induced by femtosecond laser ablation of silicon in vacuum has been studied by using spectroscopy. The spectra radiated from the plume consist of several spectral lines and continuous spectra. The spectral lines are emitted from silicon atoms and monovalent ions, while the continuous spectra are caused by recombination, bremsstrahlung, and electron-atom collisions. The spatial distributions of different spectral components vary. There are approximately two zones. The center of the first zone with a radius of approximately 0.25 mm is approximately at x = 0 and z = 0.5 mm, while the second zone is behind the first zone and approximately fan-shaped with a larger area. The ionic lines primarily radiate from the first zone, while the atomic line radiates from both zones. The continuous radiation is very strong in the first zone and becomes weak in the second zone. To understand the spatial evolution of the plume, the intensities of the three spectral components are plotted as a function of distance. An “Exponent-Allometric function” is introduced to fit the experimental data. The results indicate that there are two evolution mechanisms in the plume. One is an exponential decay component with a decay constant of approximately 0.151–0.163 mm, which is mainly attributed to recombination in the plasma plume and results in the exponential decay characteristics of ionic radiation, recombination radiation, and bremsstrahlung radiation. The other component is the allometric decay component with an allometric exponent of approximately − 1.475 to − 1.376 and caused by the expansion of the plume and responsible for the evolution of the silicon atoms. The profiles of spectral lines are effectively fitted by the Voigt function to obtain the FWHM $w_{l}$ that is caused by the Stark effect. The electron density spatial distribution is then confirmed and shows two zones. The first zone with a peak value of 3.7 × 10^18^/cm^3^ is at x = 0 and z = 0.5 mm, which is the same as the spectral intensity distribution. The second zone with a peak value of 1.1 × 10^18^/cm^3^, which is at approximately z = 1.5 mm, is arrowhead-shaped. In the first zone, the *N*_*e*_ decay profile is effectively fitted via an exponential decay function with a decay constant of 0.158 mm, which is consistent with the case of the spectral intensity distribution; therefore, the exponential decay component strongly depends on the electron density in the plasma plume. In the second zone, the nature of the arrowhead shape potentially indicates that the collision between the ambient molecules and the particles in front of the plume greatly contributes to causing an increase in the electron density, resulting in the second peak of electron density at approximately z = 1.5 mm. The experimental results and discussion in our study provide deeper insight into the spatial distribution and evolution characteristics of the fs laser-induced plume on a silicon surface, which produces a better understanding on femtosecond laser ablation of silicon and potentially can improve femtosecond laser processing technology.

## Data Availability

The datasets used and/or analyzed during the current study are available from the corresponding author on reasonable request.
